# Increased risk of chronic fatigue syndrome following burn injuries

**DOI:** 10.1186/s12967-018-1713-2

**Published:** 2018-12-05

**Authors:** Shin-Yi Tsai, Cheng-Li Lin, Shou-Chuan Shih, Cheng-Wei Hsu, Kam-Hang Leong, Chien-Feng Kuo, Chon-Fu Lio, Yu-Tien Chen, Yan-Jiun Hung, Leiyu Shi

**Affiliations:** 1 Department of Laboratory Medicine, Mackay Memorial Hospital, Taipei, Taiwan; 2 Department of Medicine; Graduate Institute of Long-Term Care, Mackay Medical College, Taipei, Taiwan; 3 Department of Health Policy and Management, Johns Hopkins University Bloomberg School of Public Health, Baltimore, United States; 4 College of Medicine, China Medical University, Taichung City, Taiwan; 5 Management Office for Health Data, China Medical University Hospital, Taichung City, Taiwan; 6 Department of Medical Research, Mackay Memorial Hospital, Taipei, Taiwan; 7 Department of Internal Medicine, Mackay Memorial Hospital, Taipei, Taiwan; 8 Institute of Infectious Disease, Mackay Memorial Hospital, Taipei, Taiwan; 9 Centro Hospitalar Conde de Sao Januario, Macao, China

**Keywords:** Burn, Thermal injury, Chronic fatigue syndrome, National health programs, Immune system diseases

## Abstract

**Background:**

The overlapping symptoms and pathophysiological similarities between burn injury and chronic fatigue syndrome (CFS) are noteworthy. Thus, this study explores the possible association between burn injury and the subsequent risk of CFS.

**Method:**

We used data from the Taiwan National Health Insurance system to address the research topic. The exposure cohort comprised of 17,204 patients with new diagnoses of burn injury. Each patient was frequency matched according to age, sex, index year, and comorbidities with four participants from the general population who did not have a history of CFS (control cohort). Cox proportional hazards regression analysis was conducted to estimate the relationship between burn injury and the risk of subsequent CFS.

**Result:**

The incidence of CFS in the exposure and control cohorts was 1.61 and 0.86 per 1000 person-years, respectively. The exposure cohort had a significantly higher overall risk of subsequent CFS than did the control cohort (adjusted hazard ratio [HR] = 1.48, 95% confidence interval [CI] = 1.41–1.56). The risk of CFS in patients with burn injury in whichever stratification (including sex, age, and comorbidity) was also higher than that of the control cohort.

**Conclusion:**

The findings from this population-based retrospective cohort study suggest that thermal injury is associated with an increased risk of subsequent CFS and provided a point of view suggesting burn injuries in sun- exposed areas such as the face and limbs had greater impact on subsequent development of CFS compared with trunk areas. In addition, extensively burned areas and visible scars were predictors of greater physiological and psychosocial that are needed to follow-up in the long run.

## Background

Chronic fatigue syndrome (CFS) is characterized by a variety of symptoms that extend beyond fatigue [1]. In 1994, Fukuda et al. defined CFS as unexplained, persistent fatigue lasting over 6 consecutive months and not improved by rest, in addition to the presence of four or more of the following symptoms [2]. Over 30 years it has numerous studies dedicated to it and verified definitions and diagnostic criteria for this disease. The diagnostic criteria (1994 CDC/Fukuda) requires a severe persistent fatigue for at least 6 months with an addition of four or more symptoms, such as unusual post-exertion fatigue, impaired memory or concentration, unrefreshing sleep, headache, muscle pain, joint pain, sore throat, and tender cervical nodes [3].

The exact cause of CFS remains under debate [4, 5]. Moreover, because CFS impacts productivity and social economy [6], identifying people at risk for CFS is imperative. We identified notable, concomitant pathophysiological features between CFS and burn injury. Patients with burn injury have high prevalence of fatigue symptoms [7, 8] and approximately 45% experience psychiatric problems, including anxiety, posttraumatic stress disorder (PTSD), and depression, even years after the incident [9]. These psychiatric conditions overlap with major symptoms of CFS such as poor sleep and chronic pain. Although the overlapping symptoms and pathophysiological similarities between burn injury and CFS are noteworthy, the exact relationship between the two has yet to be established. The aim of our population-based prospective study was to analyze the hazard ratios (HRs) and cumulative incidence rates of CFS between patients with and without burn injuries by using the National Health Insurance Research Database (NHIRD) of Taiwan.

## Methods

### Data source

This was a retrospective, population-based cohort study conducted through claims data from Taiwan’s National Health Insurance (NHI) program that provides coverage for over 99% of the 23 million Taiwan residents since March 1, 1995 (Database NHIR. Taiwan, http://nhird.nhri.org.tw/en/index.html). The National Health Research Institutes (NHRI) has collected health claims data in a de-identified format and established the NHIRD. The Longitudinal Health Insurance Research Database 2000 (LHID2000), a subset of the NHIRD, contains claims data for one million beneficiaries randomly sampled from all insurance enrollees in the NHIRD. The details of LHID2000 have been previously described [24]. This study was approved by the Institutional Review Board of China Medical University (CMUH-104-REC2-115) and the Institutional Review Board of MacKay Memories Hospital (16MMHIS074).

### Sampled participants

The International Classification of Disease, Ninth Revision, Clinical Modification (ICD-9-CM) was used to define diagnostic disease status. Patients aged 20 years or older with newly diagnosed burn injury (ICD-9-CM codes 940, 941, 942, 943, 944, 945, 946, 947, 948) between 2000 and 2010 were identified and recruited in the burn cohort. The date of burn diagnosis was defined as the index date. Patients without a history of burn injury were randomly selected from LHID2000 and included in the nonburn cohort. To increase the statistical power, we used fourfold more patients in the nonburn cohort matched by age (every 5 years of age), sex, and index year for each burn case. Patients in both cohorts who were aged younger than 20 years, had a history of CFS (ICD-9-CM code 780.71) before the index date, or had incomplete information on age or sex were excluded.

### Outcome and comorbidity

Each patient was followed until one of the following outcomes occurred: diagnosis of CFS, death of the patient, patient withdrawal from the NHI program, or the end of the study (December 31, 2011). Baseline comorbidities for CFS were included as follows: diabetes (ICD-9-CM code 250), obesity (ICD-9-CM code 278.0), renal disease (RD; ICD-9-CM codes 580–589), rheumatoid arthritis (RA; ICD-9-CM code 714), HIV (ICD-9-CM code 042), malignancy (ICD-9-CM codes 140–149, 150–159, 160–165, 170–172, 174–175, 179–189, 190–199, 200–208, and 235–238), depression (ICD-9-CM codes 296.2, 296.3, 300.4, and 311), anxiety (ICD-9-CM code 300.00), sleep disorder (ICD-9 codes 307.4 and 780.5), and irritable bowel syndrome (ICD-9-CM code 564.1).

## Statistical analysis

We compared differences in demographic factors and comorbidities between the cohorts with and without burn by using the Chi squared test. The Kaplan–Meier analysis was used to compare the cumulative incidence of CFS in the burn and nonburn cohorts, with significance based on the log-rank test. We compared the incidence density rate of CFS between the cohorts stratified by sex, age, and comorbidity. Univariate and multivariate Cox proportional hazards analyses were used to investigate the association between burn and the risk of developing CFS, and were adjusted for age, sex, and comorbidities of diabetes, obesity, RD, RA, HIV, malignancy, depression, anxiety, sleep disorder, and irritable bowel syndrome. A two-tailed p value of < 0.05 was considered statistically significant. SAS software version 9.4 (SAS Institute Inc., Carey, NC) was used for data analyses.

## Results

**Fig. 1:**
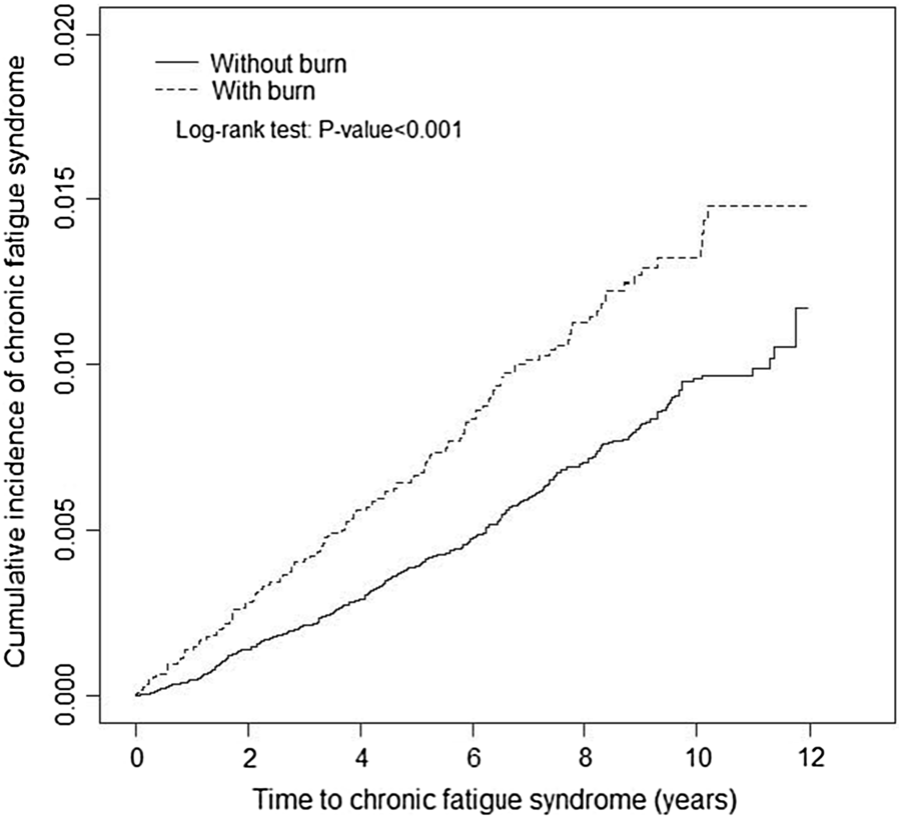
Cumulative incidence of chronic fatigue syndrome in patients with and without burn injury using the Kaplan–Meier method

A total of 17,204 patients were included in the burn cohort and 68,812 in the nonburn cohort (Table 1). The majority of patients were women (52.2%) and ≤ 64 years old (61.8%) in both cohorts. The patients in the burn cohort tended to have a higher prevalence of diabetes, obesity, RD, depression, anxiety, sleep disorder, and irritable bowel syndrome than did those in the nonburn cohort, the prevalence of HIV was higher in nonburn cohort, no significant difference (p-value < 0.05) was showed in malignant and RA between the two cohorts. In addition, the mean follow-up periods for the burn and nonburn cohorts were 5.85 and 5.89 years, respectively. The cumulative incidence of CFS was significantly greater for patients in the burn cohort, compared with patients in the nonburn cohort (log-rank test, p < 0.001; Fig. 1). The overall incidence density rate of CFS was 61% higher in the burn cohort than in the nonburn cohort (1.39 vs. 0.86 per 1000 person-years), with an adjusted HR (aHR) of 1.48 (95% CI, 1.41–1.56) in the following 12 years (Table 2). The incidence density rates and HRs for CFS, as stratified by sex, age, and comorbidity, were all higher in the burn cohort than in the nonburn cohort. Among the patients in the nonburn cohort, compared with those without comorbidities, those with only sleep disorder had the highest risk of CFS (aHR, 2.07; 95% CI, 1.90–2.25), followed by those with only depression (aHR, 1.80; 95% CI, 1.14–2.85), and then those with only anxiety (aHR, 1.16; 95% CI, 1.02–1.31). Moreover, compared with the nonburn patients without these comorbidities, patients in the burn cohort with two or more comorbidities had a significantly increased risk of CFS (aHR, 2.93; 95% CI, 2.70–3.18), followed by those with any one comorbidity (aHR, 2.28; 95% CI, 2.09–2.49; Table 3).

**Table 1:** Demographic characteristics and comorbidities in patients with and without burn injury

Variable	Burn		*p*-value
	No	Yes	
	N = 68,812	N = 17,204	
Sex	n(%)	n(%)	0.99
Female	35,896(52.2)	8974(52.2)	
Male	32,916(47.8)	8230(47.8)	
Age, mean(SD)	45.4(17.3)	45.6(17.2)	0.14^*^
Age group			0.99
≤ 49	21,576(31.4)	5394(31.4)	
50-64	20,908(30.4)	5227(30.4)	
65+	26,328(38.3)	6583(38.3)	
Comorbidity			
Diabetes	4299(6.25)	1701(9.89)	<0.001
Obesity	776(1.13)	266(1.55)	<0.001
Renal disease	3394(4.93)	1201(6.98)	<0.001
Rheumatoid arthritis	112(0.16)	38(0.22)	0.10
HIV	32(0.05)	2(0.01)	0.02
Malignancy	1374(2.00)	319(1.85)	0.23
Depression	2556(3.71)	1135(6.60)	<0.001
Anxiety	8552(12.4)	3137(18.2)	<0.001
Sleep disorder	10,049(14.6)	3731(21.7)	<0.001
Irritable bowel syndrome	2611(3.79)	874(5.08)	<0.001

P-values from Chi square test, except where noted differently

*Two sample t-test

**Table 2:** Comparison of incidence and hazard ratio of chronic fatigue syndrome stratified by sex, age and comorbidity between patients with and without burn

Variable	Burn						Crude HR (95% CI)	Adjusted HR^†^ (95% CI)
	No			Yes				
	Event	PY	Rate^#^	Event	PY	Rate^#^		
All	349	405,204	0.86	140	100,663	1.39	1.61(1.53, 1.70)***	1.48(1.41, 1.56)***
Sex								
Female	192	213,599	0.90	78	53,268	1.46	1.63(1.52, 1.75)***	1.46(1.36, 1.56)***
Male	157	191,605	0.82	62	47,395	1.31	1.60(1.48, 1.72)***	1.41(1.31, 1.51)***
Age group								
≤ 49	63	131,799	0.48	21	33,051	0.64	1.33(1.21, 1.47)***	1.24(1.13, 1.37)***
50–65	100	132,948	0.75	36	33,088	1.09	1.45(1.32, 1.59)***	1.29(1.18, 1.42)***
65+	186	140,457	1.32	83	34,524	2.40	1.82(1.68, 1.96)***	1.65(1.53, 1.78)***
Comorbidity^‡^								
No	205	302,276	0.68	58	66,009	0.88	1.30(1.21, 1.39)***	1.35(1.26, 1.44)***
Yes	144	102,928	1.40	82	34,654	2.37	1.69(1.56, 1.84)***	1.72(1.59, 1.87)***

*P*-values form the Cox proportional model; ***p < 0.001

^#^Incidence rate per 1000 person-years. ^†^Multivariate analysis that includes the following variables: age, sex, and comorbidities of diabetes, obesity, renal disease, rheumatoid arthritis, HIV, malignancy, depression, anxiety, sleep disorder and irritable bowel syndrome. ‡Patients with any one of the comorbidities diabetes, obesity, renal disease, rheumatoid arthritis, HIV, malignancy, depression, anxiety, sleep disorder and irritable bowel syndrome were classified as the comorbidity group

*HR*  hazard ratio, *PY* patient-year

**Table 3:** Joint effects for chronic fatigue syndrome in burn injury and chronic fatigue syndrome-associated risk factors

Variable	N	No. of events	Rate^#^	Adjusted HR^†^	95% CI
None	48,381	205	0.67	1	(Reference)
Only burn	10,417	58	0.87	1.34	(1.25, 1.44)***
Only diabetes	1919	11	1.07	0.99	(0.86, 1.14)
Only obesity	426	1	0.56	0.83	(0.53, 1.32)
Only renal disease	1291	6	0.74	0.76	(0.63, 1.00)
Only rheumatoid arthritis	49	0	0.00	–	–
Only HIV	23	0	0.00	–	–
Only malignancy	605	0	0.00	–	–
Only depression	155	1	1.29	1.80	(1.14, 2.85)*
Only anxiety	2428	15	1.03	1.16	(1.02, 1.31)*
Only sleep disorder	4102	34	1.73	2.07	(1.90, 2.25)***
Only irritable bowel syndrome	841	4	0.85	1.01	(0.80,1.27)
Burn with any one comorbidity	3383	36	1.96	2.28	(2.09, 2.49)***
Burn with any two comorbidity	3404	46	2.82	2.93	(2.70, 3.18)***

Rate^#^, per 1000 person-year

Adjusted HR^†^: multivariable analysis including age, and sex

* P < .05

** P < .01

*** P < .001

Burn patients with total body surface area (TBSA) of burn between 20% and 50% had higher risk of CFS (aHR, 3.43; 95% CI, 2.67–4.40), compared with the patients in the nonburn cohort (Table 4). Similar results to a lesser extent were observed among patients with TBSA of burn < 20% (aHR, 2.11; 95% CI, 1.92–2.31). Compared with the patients without burn, burn patients with injured parts of the eye, adnexa of the eye, face, head, and neck had the highest risk of CFS (aHR, 2.24; 95% CI, 1.92–2.61), followed by those who sustained injuries in the upper and lower limbs (aHR, 1.84; 95% CI, 1.54–2.20), and lastly followed by those who sustained injuries in the wrist and hand (aHR, 1.43; 95% CI, 1.35–1.53).

**Table 4:** Comparisons of Incidence, and Hazard Ratios of chronic fatigue syndrome by subtypes of burn

Variables	N	Event	Rate^#^	Crude HR (95% CI)	Adjusted HR^†^ (95% CI)
Non-burn (ICD-9-CM)	68,812	349	0.86	1(Reference)	1(Reference)
Burn					
Excluded the following codes	13,845	105	1.29	1.50(1.42, 1.59)***	1.49(1.41, 1.57)***
TBSA % < 20% (ICD-9-CM 948.0, 948.1).	3072	31	1.74	2.02(1.83, 2.21)***	2.11(1.92, 2.31)***
TBSA % = 20% to 50% (ICD-9-CM 948.2 ~ , 948.4),	234	4	2.78	3.22(2.50, 4.15)***	3.43(2.67, 4.40)***
TBSA % > 50% (ICD-9-CM 948.5 ~ 948.9)	53	0	0.00	–	–
Burn confined to eye and adnexa & Burn of face, head and neck (ICD-9-CM 940, 941)	906	11	2.30	2.67(2.29, 3.11)***	2.24(1.92, 2.61)***
Burn of trunk (ICD-9-CM 942)	765	4	0.84	0.98(0.76, 1.26)	0.95(0.74, 1.22)
Burn of upper limb, except wrist and hand & Burn of lower limb (ICD-9-CM 943, 945)	745	8	1.96	2.27(1.90, 2.72)***	1.84(1.54, 2.20)***
Burn of wrist and hand (ICD-9-CM 944)	10,375	80	1.35	1.56(1.47, 1.66)***	1.43(1.35, 1.53)***
Others	4413	37	1.34	1.56(1.43, 1.70)***	1.48(1.35, 1.61)***

Rate^#^, incidence rate, per 1000 person-years; Crude HR, crude hazard ratio

Adjusted HR^†^: multivariate analysis including age, sex, and comorbidities of diabetes, obesity, renal disease, rheumatoid arthritis, HIV, malignancy, depression, anxiety, sleep disorder and irritable bowel syndrome

***p < 0.001

TBSA = Total burn surface area

## Discussion

Even with the latest criteria, diagnosing CFS remains as challenging as ever for physicians. 60% of patients remain undiagnosed [3]. The difficulty of diagnosing CFS not only attributes to the nonspecific symptoms but possibly to the lack of familiarity for general physicians on the topic. It is reported that less than one-third of medical schools discuss CFS in their curriculum and only 40% of textbooks include information on the disorder [10]. In addition, CFS also causes great loss of social economy due to the impartment of patients’ labor force, which have been estimated at $17 to $24 billion annually [11]. Patients with CFS who need household care can suffer worse economy pressure due to the higher medical costs.

Our results show that the cumulative incidence of CFS was significantly higher in the burn cohort, compared with the non-burn cohort. This indicates that burn injury is a risk factor to developing CFS. The results could be illustrated by our hypothesis in that burn patients who suffer from infections are more likely to develop CFS compared with control groups.

To the best of our knowledge, it is the first study to establish the relationship between patients with burn injury and CFS occurrence, based on nationwide data in an Asian population. Our results demonstrate that burn injuries are a risk for subsequent CFS. Total body surface area (TBSA) of burn is known as a potential factor that affects the post-burn recovery. An earlier study showed that the most severely burn injured patients experienced poorer recovery in physical and mental health status [12].

The mechanism of chronic fatigue syndrome is still not clear yet. Numerous evidence imply of the association between the hypofunction of HPA axis and sympathetic adrenal medulla (SAM) system. The responsivity of HPA axis is attenuated in chronic fatigue syndrome with the weakened cortisol response to common daily stressors [13]. One study revealed that merely one-third Myalgic Encephalomyelitis (ME)/CFS patients were observed and the incidence of HPA axis hypofunction was increased along with the severity of CFS [14]. The evidence indicated that the hypo-function of HPA axis was a result rather than a cause.

Our result showed that there was a higher risk of CFS in thermal injury patients, so we searched for the evidence of HPA axis disturbance after burn trauma. After a thermal injury, numerous proinflammatory cytokines lead to the Systemic inflammatory responsive syndrome(SIRS) as the first phase of thermal injury [15]. The interactions between Proinflammatory cytokines (e.g., IL-1, IL-6, IL-17, TNFα), reduced neurotrophic factors, elevated oxidative stress and dysregulation of HPA-axis are well documented and these relationships have become established and published hypothesis. Some hypothesis involve further development to several psychotic diseases including major depression and schizophrenia [16, 17]. We speculated that the disturbance of HPA axis happens in the beginning of the thermal injury. Furthermore, patients in the burn cohort with two or more comorbidities have a significantly increased risk of CFS. This indicates that we should not only treat patients their thermal wounds but also treat their underlying diseases or newly evolved clinical problems to reduce the risk of developing CFS.

The mental status of patients also should be a concern as HPA regulates daily stress. Due to the irreversible nature of the dysfunction and disfigurement, the higher the TBSA the higher the likelihood to develop massive stress, impairing patient’s mental status and dysregulating the HPA axis. More than 90% of burn patients suffered from acute stress and more than 45% developed PTSD in a year [18]. In fact, chronic fatigue syndrome shares several similar features with post-traumatic stress disorder in terms of the attenuation of HPA axis response [19]. As a higher TBSA is related to anxiety and higher incidence of PTSD, we can speculate that a higher TBSA affects the HPA axis and ultimately leads to higher incident rate of both PTSD and CFS.

The higher risk between CFS and the thermal injury patient with higher TBSA is understandable; it is noteworthy that in our study the incidence rate in patients with TBSA level lower than 20% was still statistically significant. This group of patients is easily neglected due to either a quicker recovery or needing fewer follow-ups. Worse, with chronic malaise and nonspecific symptoms CFS is frequently delayed in diagnosis [3]. Our study introduced the insight that we should inform both the physician and the patients with any level of TBSA to be aware of first, the patient’s physical state and second, appropriate and subsequent referrals.

Our findings provide a perspective suggesting that burn injuries occurring in the most sun-exposed areas of the body, such as the face and limbs, have greater impact on subsequent development of CFS when compared with those occurring in skin areas of the torso. Deformity of the face and hands and disabilities are often prevalent in patients with large TBSA of burns; consequently, depression, anxiety disorder, and PTSD were also reported in this population [20]. A recent study demonstrated that self-criticism is associated with increased stress and reduced hypothalamic–pituitary–adrenal (HPA)-axis reactivity [21]. These findings suggest that depression caused by the disfigurement of sun-exposed areas may affect the function of HPA axis and lead to a higher risk of subsequent CFS, compared with disfigurement in the torso area. Consequently, early identification and social support for relieving the stress is important for these group of patients. Indeed, psychosocial interventions (such as stress management skills) were shown to improve the symptoms and severity of CFS [22].

Evidence supports that increased oxidative and nitrosative stress is a possible etiology in CFS/ME patients. Increased level of oxidative and nitrosative biomarkers were found in CFS patients. Nitric oxide (NO) is a cellular signal that participates in the redox pathway and plays a role in regulation of HPA axis by blunting nicotine- induced corticosterone and ACTH. Oxidative stress (free radicals), which cause damage to healthy cells and mitochondria dysfunction, is an essential factor leading to fatigue. Several studies have reported that there is a positive correlation between oxidative stress measurements and symptom severity [17]. Major oxidative stress with increased serum level of reactive oxygen species (ROS) was observed in thermal injury patients with SIRS [23]. These ROS originates from mitochondrial electron transport, peroxisomal ß-oxidation and phagocytic NADPH, and contributes to detoxifying acute doses of toxins and defending against environmental pathogens. Thus, massive ROS leads to the production of hydroxyl radical (OH) which is responsible for oxidative damage to cellular macromolecules and cellular dysfunction [24]. CFS was reported to be associated with very long lncRNA (long non-coding RNA) expression signatures, which is related to oxidative stress, chronic viral infection and hypoxemia. LncRNAs are defined as RNAs that are more than 200 nucleotides long, which do not encode proteins. Through RNA–DNA, RNA–RNA, or RNA–protein interactions, lncRNA can affect different stages of gene regulation in immune response and neurologic processes, thus potentially playing a role in ME/CFS [25]. One study identified a 256-peptide signature that separates ME/CFS samples from healthy controls, suggesting that the hypothesis of immune dysfunction merits further investigation and work towards a clinically meaningful diagnostic biomarker [26].

Our study has several limitations. Because we did not recruit healthy people as the control group, we might have underestimated the risk of CFS, despite adjusting for CFS-related comorbidities. Furthermore, CFS diagnosis can overlap with that of depression, depression is associated with aggravated pain and insomnia, not with new onset of pain, migraine, irritable bowel, or autonomic dysfunction [27]. and patients with depression are more likely to develop chronic pain and fatigue-related conditions later in life [28]. Depression is also a common comorbidity in patients with burn injury. Because of the unavailability of biomarkers for definite diagnosis of CFS, we cannot rule out the possibility of misdiagnosis of the population with depression. However, because of the low incidence of depression in our burn cohort, it is expected to have only a limited impact on our findings. In addition, the NHI system was operated by the government since 1995. Although the diagnoses of burn and CFS were based on the ICD-9-CM code determined by clinical physicians, medical reimbursement specialists should examine all insurance claims. Consequently, the diagnoses and codes for burn and CFS should be reliable in our study. Future study can be more focused on demonstrating the mechanism of CFS following burn injury. Studies focusing on clinical intervention of potential treatments (probiotics, psychological monitoring) and the clinical outcome of those treatments are expected in the future to prevent CFS after burn injury.

## Conclusion

In summary, the clinical implications of our study highlight the need for burn victims and their physicians to be aware of the subsequent risk of CFS. Appropriate preventive steps may be vital to promote recovery and enhance health-related quality of life.

## Abbreviations

CFS: chronic fatigue syndrome
HPA axis: hypothalamic–pituitary–adrenal axis
NHIRD: National Health Insurance Research Database
LHID: Longitudinal Health Insurance Database
SD: standard deviation
HRs: hazard ratios
CIs: confidence intervals
PICs: proinflammatory cytokines
ACTH: adrenocorticotropic hormone
TBSA: total burn surface area
SIRS: systemic inflammatory response syndrome
TNFα: tumor necrosis factor α
IL-1: Interleukin-1
RD: renal disease
RA: rheumatoid arthritis
Anti-LPS: anti-lipopolysaccharide
